# Gastric Intussusceptions in a Red Corn Snake* (Pantherophis guttatus)* Associated with Cryptosporidiosis

**DOI:** 10.1155/2017/4270904

**Published:** 2017-05-07

**Authors:** Marjorie Bercier, Whitney Zoll, Justin F. Rosenberg, Robson Giglio, Lenice McCoy, William L. Castleman, Matthew D. Johnson, Darryl J. Heard

**Affiliations:** ^1^Department of Small Animal Clinical Sciences, College of Veterinary Medicine, University of Florida, 2015 SW 16th Avenue, Gainesville, FL 32610, USA; ^2^Department of Infectious Diseases and Pathology, College of Veterinary Medicine, University of Florida, 2015 SW 16th Avenue, Gainesville, FL 32610, USA; ^3^Department of Large Animal Clinical Sciences, College of Veterinary Medicine, University of Florida, 2015 SW 16th Avenue, Gainesville, FL 32610, USA

## Abstract

A 3-year-old female red corn snake* (Pantherophis guttatus)* was presented for a three-week history of anorexia and decreased defecations. On physical examination, a soft midbody intracoelomic swelling was palpated. Transcutaneous coelomic ultrasound revealed a target-like mass on a transverse section of the stomach, suggesting the presence of a gastrointestinal intussusception. On exploratory coeliotomy, a double compounded esophagogastric and gastroduodenal intussusception was diagnosed and reduced surgically. A gastropexy was also performed to prevent recurrence. On histopathology, the gastric glandular mucosa showed moderate to marked proliferation. Diffusely lining the luminal surface of glandular epithelium and free within the lumen were a myriad of protozoa consistent with* Cryptosporidium* sp. A diagnosis of chronic proliferative gastritis due to* Cryptosporidium* sp. was made based on these findings. Intussusceptions are rare in reptiles and are infrequently reported in snakes. This is the first report of a double compounded intussusception in a nonmammalian species and the first report of an intussusception involving the stomach in a snake with gastritis due to* Cryptosporidium* sp.

## 1. Introduction

*Cryptosporidium* spp. is an apicomplexan parasite that is known to affect mammals including humans, birds, fishes, amphibians, and reptiles [1, 2]. Infections have been reported in more than 80 species of reptiles, including snakes, lizards, and tortoises [1]. Two main* Cryptosporidium* spp. are frequently reported in reptiles:* Cryptosporidium serpentis* and* Cryptosporidium varanii*.* C. serpentis* is highly pathogenic in snakes and causes chronic hypertrophic gastritis. Clinical signs include regurgitation, anorexia, muscle wasting, midbody swelling, and death [2, 3]. The aim of this report is to describe the diagnosis and surgical treatment of a double compounded esophagogastric and gastroduodenal intussusception in a red corn snake* (Pantherophis guttatus)* with* Cryptosporidium* sp.-associated gastritis.

## 2. Case Report

An approximately 3-year-old, intact female, 260 g red corn snake* (Pantherophis guttatus)* was evaluated by the Zoological Medicine Service, University of Florida, for a midbody swelling and a three-week history of anorexia. The snake was purchased from a breeder 2.5 years priorly and did not have any previous health concerns. The reptile was kept in a tank with aspen bedding, in a room kept at 25°C throughout the year. During winter, supplemental heating was offered in one end of the tank using a heat lamp. Temperature and humidity in the enclosure were not monitored. The snake was fed a frozen-thawed adult mouse once weekly. During the feedings, the snake was transferred to a different container without any bedding, where it was left with the food item until consumed. The owner reported that the snake normally defecated regularly, but that for the past three to four weeks the stools had been dryer, smaller, and less frequent. The snake remained active at home and displayed a normal behaviour. The owner also had a wild caught, apparently healthy grey ratsnake* (Pantherophis spiloides)* kept in a separate tank.

**Figure 1:**
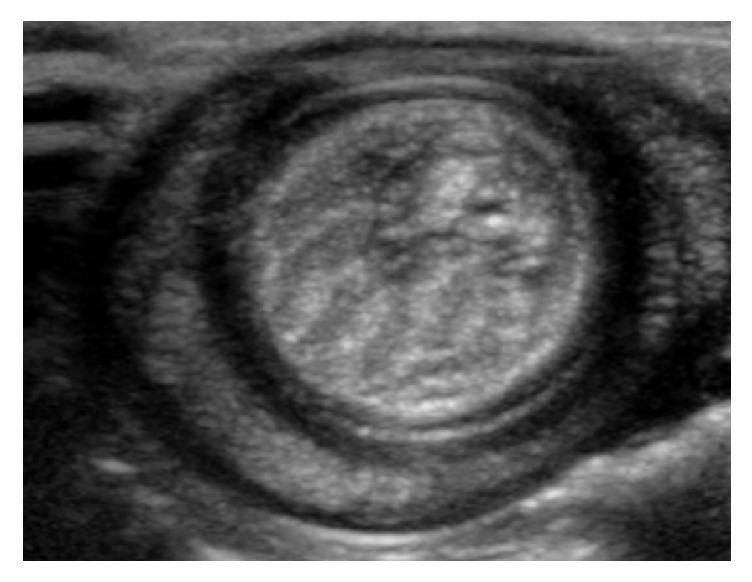
Short axis ultrasonographic image of the stomach demonstrating the typical target-like (multilayering) appearance seen in cases of intussusception.

During physical examination, the red corn snake was quiet, alert, and responsive, and appeared in good body condition. A soft, ventral, intracoelomic swelling (6 × 4 cm) was palpable approximately at midbody and elicited discomfort to the patient when manipulated. Ultrasonography (Philips iU22 ultrasound machine, Philips Medical Systems, Bothell, WA 98021, USA), through multifrequency linear transducers, revealed a cylindrical structure lateral to the caudal liver, coursing caudally and medially to reside in proximity to the stomach. At this location, the segment thought to represent the stomach was markedly and focally fluid dilated. Caudally, from this region of dilation, an additional cylindrical segment surrounded those described previously, resulting in a concentric ring appearance (Figure 1). The central component of the concentric ring demonstrated blood flow, using color Doppler. Based on these findings, a gastrointestinal intussusception was suspected and surgery was recommended.

**Figure 2:**
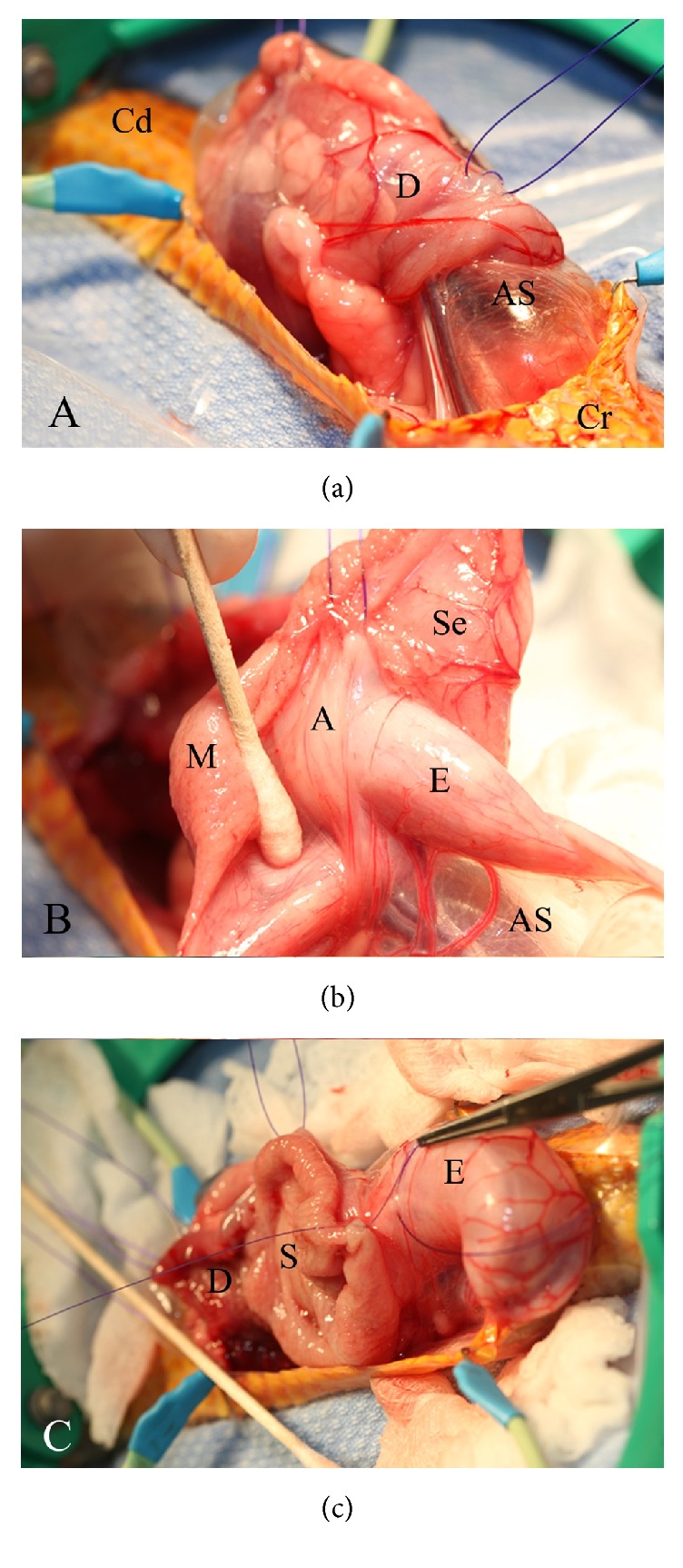
(a) Gastroduodenal intussusception. The duodenum (D) and air sac (AS) are readily visible at the surgical site. The cranial (Cr) and caudal (Cd) directions of the snake are identified. (b) Esophagogastric intussusception. After the gastrotomy, adhesions (A) are identified between the esophagus (E) and the stomach's serosa (Se). The hypertrophied gastric mucosa (M) is visible. (c) Reduction of the double compounded intussusception. Depicted here are the esophagus (E), stomach (S), and duodenum (D) in their normal anatomic position.

The snake was sedated with 0.1 mg/kg of body weight (BW) of hydromorphone (West-Ward, Eatontown, NJ 07724, USA) administered intramuscularly. Intubation with a 14 G catheter was possible following the sedation and the animal was maintained on 1-2% isoflurane (Piramal Healthcare Limited, Andhra Pradesh 502321, India) in a mixture of oxygen and nitrous oxide (1 L/min of each). The patient received positive intermittent manual ventilation at a rate of four breaths per minute. The patient was placed in left lateral recumbency, and the surgical site was prepped aseptically. A 10 cm incision was made two scale rows dorsal to the ventral scales. The incision followed along the edges of the scales, forming a scalloped pattern. The underlying muscle layers were incised at the end of the ribs. A Lonestar retractor (Jorgensen Labs Inc., Loveland, CO 80538, USA) was used to improve visualization of the coelomic cavity. After manipulation of the tissues, a gastrotomy was made laterally and spanned most of the length of the stomach and into the duodenum approximately 2 cm. The esophagus was observed to be intussuscepted into the stomach (Figure 2(b)), and then this combination was further invaginated into the duodenum (Figure 2(a)) forming a second intussusception. A diagnosis of double compounded esophagogastric and gastroduodenal intussusception was made. The stomach was extracted from the duodenum and then the esophagus was extracted from the stomach. There were adhesions formed between the esophagus and stomach that were bluntly dissected in order to extract the esophagus from the stomach (Figure 2(c)). Grossly, the gastric longitudinal rugae appeared hypertrophied. Samples of gastric mucosa were placed into 10% buffered neutral formalin for histopathologic evaluation. The stomach was closed in two layers with 3-0 PDS (Ethicon LLC, Cincinnati, OH 45242, USA) with a Lembert pattern in the mucosa/submucosa layer, followed by a simple continuous pattern for the serosal muscularis layer. Due to the nature of the intussusception, the incision in the duodenum had a transverse as well as a longitudinal component. The transverse incision in the duodenum was closed with four simple interrupted sutures using 4-0 PDS (Ethicon LLC, Cincinnati, OH 45242, USA). The longitudinal component was closed with a simple continuous pattern using 4-0 PDS. A gastropexy via interrupted circumcostal sutures was performed with 3-0 PDS to prevent recurrence of the intussusceptions. The body wall was closed routinely. The muscle layer was closed with 3-0 PDS in a simple continuous manner. The integument was closed with nine horizontal mattress and one simple interrupted sutures to cause an eversion of the scales using 3-0 PDS.

The patient was discharged the following day. Treatments included famotidine (Mylan Institutional LLC, Rockford, IL 661103, USA) at 0.026 mg/kg BW SC q48h, ceftazidime (Hospira Worldwide Inc., Lake Forest, IL 60045, USA) at 22 mg/kg BW SC q72h, and meloxicam (Putney Inc., Portland, ME 04101, USA) at 0.1 mg/kg BW SC q48h. The snake was not fed for two weeks before reexamination. The owner provided a supplemental heat lamp in the tank to have a warm basking spot and a cooler temperate area. All bedding was removed from the enclosure and only clean newspaper or paper towels were used as a substrate.

**Figure 3:**
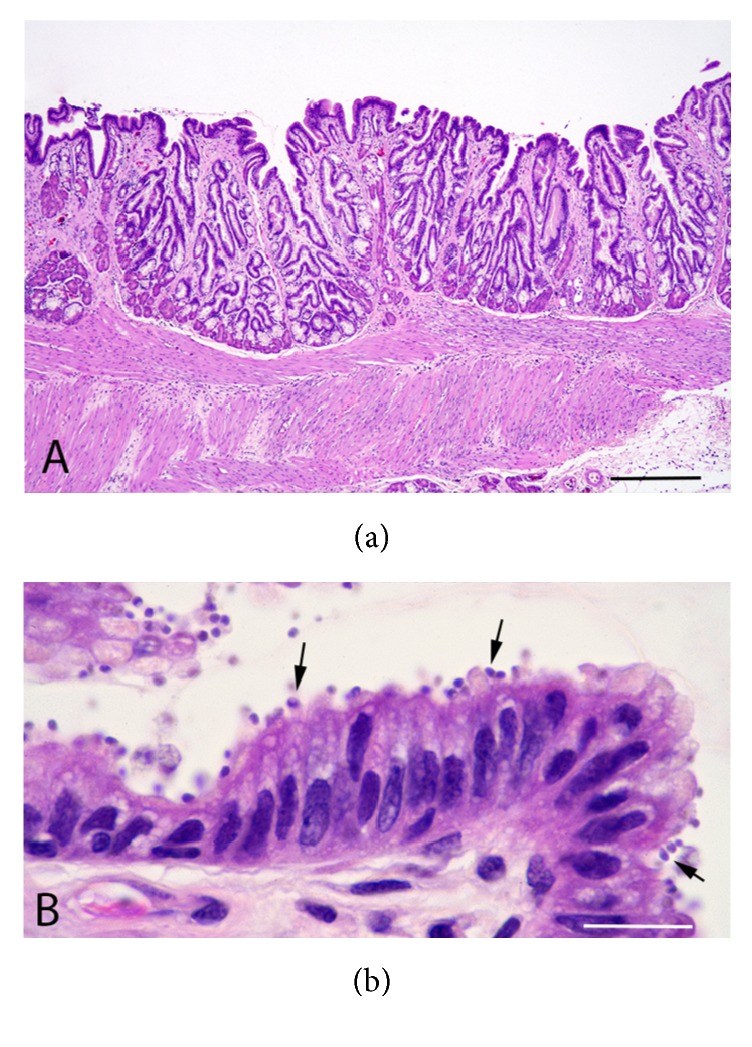
(a) Low power magnification image of chronically inflamed stomach with hyperplasia of surface and glandular epithelium. H&E stain; bar = 142 *μ*m. (b) High power magnification image of hyperplastic gastric glandular and surface epithelium with protozoa (arrows) closely associated with the apical surface membranes of the epithelial cells. Other protozoa are free in the lumen. H&E, bar = 14 *μ*m.

In histologic sections of the stomach, epithelium on the gastric surface and in gastric glands was hyperplastic (Figure 3(a)). Dilation of the mucosal glands and fibrosis of the lamina propria were also evident. Myriad protozoa that were 2 *μ*m in diameter, eosinophilic to basophilic, and periodic-acid-Schiff- (PAS-) positive and that had variably distinct 0.5–2 *μ*m basophilic nuclei were closely associated with the apical epithelial surfaces as well as being free within the lumen (Figure 3(b)). Small numbers of heterophils, lymphocytes, and plasma cells were in the lamina propria. A diagnosis of chronic proliferative gastritis due to* Cryptosporidium* sp. was made based on these findings.

Two weeks postoperatively, the snake was recovering well. The surgical site was clean, well apposed, and free of any discharge. The ventral surface associated with the incision was mildly distended and the scales appeared slightly dull. The owner had no concerns at that time, and the snake maintained BW. All medications were discontinued and the snake was tube-fed 2.5 mL of carnivore care (Oxbow Animal Health, Murdoch, NE 68407, USA) slurry to help encourage normal gastrointestinal movements and to administer a small first meal. The owner was allowed to start refeeding the snake smaller prey items such as pinkies every five days for 1 month, and then increasing to one hopper every 5 days for another month, followed by adult mice once weekly as the preoperative husbandry practices.

At four weeks after surgery, the skin sutures were removed. A brief ultrasound exam was performed and did not show any obvious abnormalities around the stomach. The owner reported one abnormal bowel movement, described as diarrhea, that occurred four to five days after feeding the first pinkie mouse. The owner also reported that the snake had a normal shed two weeks priorly without any complications. The animal had lost 30 g (approximately 12% of BW) since initial presentation, but this was expected due to the current feeding schedule. A gastric wash was performed and submitted for polymerase chain reaction (PCR) to speciate the* Cryptosporidium* present in the stomach. However, there were no organisms in the sample, and the PCR came back negative.

Unfortunately, at 15 months after surgery, the snake was found deceased in its enclosure. The owner reports that the snake would have intermittent episodes of regurgitation when it was fed adult mice but seemed to tolerate eating smaller food items without difficulties. The snake behaved normally, remained with a good appetite, and did not have other clinical signs until the day it was found dead. The cause of the regurgitations is unknown, but worsening of the chronic* Cryptosporidium* sp.-associated gastritis is suspected. The snake was not submitted for postmortem examination.

## 3. Discussion

An intussusception is an invagination of one segment (intussusceptum) of intestine into another (intussuscipiens). Intussuscepta are usually orad to the intussuscipiens (anterograde intussusception), but can also be aborad (retrograde intussusception). A retrospective study in domestic dogs and cats revealed that inflammatory disease and neoplasia were the most common causes of intestinal intussusceptions, respectively [4]. Intussusception has been infrequently described in reptiles [5]. It has been associated with monofilament fishing line ingestion in sea turtles [6, 7]. In a report investigating gastrointestinal tract lesions in sea turtles, seven out of 136 animals had intestinal intussusceptions, sometimes leading to necrosis of the intestinal mucosa [7]. An ileocolic intussusception has been reported in a pine snake* (Pituophis melanoleucus)*, who died shortly after a permanent ileostomy was performed [8]. Gastric intussusceptions have not previously been reported in reptiles. The* Cryptosporidium* sp.-associated gastritis likely contributed to the development of this snake's intussusceptions, in a similar fashion to what inflammatory diseases do in dogs.

In domestic dogs, transcutaneous abdominal ultrasonography is a highly sensitive (100%), specific (97.8%), and accurate (98.4%) method of diagnosis of intestinal intussusception, characterized as a target-like mass visible on transverse sections [9]. Due to their unique anatomy, characterized by their limbless, tubular, and elongated bodies, intussusception appears to be readily palpable in snakes. However, such finding is not specific and other differentials should be ruled out. Transcutaneous coelomic ultrasonography was a useful confirmatory diagnostic tool in this case, revealing the typical concentric rings in the transverse section of the stomach.

There is discrepancy in the literature regarding the nomenclature of complex intussusceptions. As described here, a double compounded intussusception is a combination of two anterograde intussusceptions at a given location [10]. A double intussusception (DI) involves two distinct intussusceptions at separate points of the bowel [10]. In the veterinary literature, the term DI has been used interchangeably for both entities. DI has been described in dogs [11–15] and cats [16], mostly of young age.

Two main* Cryptosporidium* spp. are frequently reported in reptiles.* C. serpentis* is a common pathogen of snakes and is known to cause hypertrophic gastritis.* C. varanii* (previously known as* Cryptosporidium saurophilum*) is most commonly reported in lizards; however it has also been seen in snakes [17–19].* Cryptosporidium *sp.-associated enteritis without evidence of gastritis has been reported in snakes, but the species of the causative agent was not determined [20]. Coinfection with an adenovirus likely increased one snake's susceptibility to cryptosporidiosis by creating immunodeficiency [21]. Other factors facilitating susceptibility of snakes to* Cryptosporidium* sp. gastritis are believed to be stress of captivity, translocations, and reproduction [21].

Clinical signs of* C. serpentis* gastritis include regurgitation, anorexia, muscle wasting, midbody swelling, and death [2, 3]. Diagnosis of* C. serpentis* in snakes can be made by acid-fast staining or PCR on fecal samples, gastric lavage samples, regurgitated material, or gastric biopsies [3, 19]. Gross appearance of the gastric mucosa of affected snakes includes focal or diffuse hyperemia, cobblestoned appearance, thickened mucosa, lack of rugal folds, accentuation of longitudinal ridges, and edema [2, 3, 21]. These changes can result in apparent narrowing of gastric lumen. On histopathology, chronic gastritis is characterized by proliferation, increased tortuosity and mucoid metaplasia of the pyloric mucosal glands, fibrosis around the glands, hyperplasia of mucosal lining cells, and infiltration of heterophils and lymphocytes in the lamina propria [3]. Intralesional and intraluminal* Cryptosporidium* sp. are often visible [2, 3, 21]. There is no known effective treatment for* Cryptosporidium* sp. infections in snakes. Savanna monitors have been successfully treated for cryptosporidiosis with gastric administration of hyperimmune bovine colostrum, but this treatment only decreased the parasite load and did not completely clear infection in snakes and leopard geckos [22–24]. For this reason, no treatment was implemented for cryptosporidiosis in this case.

This is the first report of a double compounded intussusception in a reptile, and the first report of an intussusception involving the stomach in a snake with gastritis due to* Cryptosporidium* sp. Transcutaneous coelomic ultrasonography was helpful in the diagnosis of the condition. Surgical reduction of the intussusceptions and gastropexy resulted in successful resolution of all clinical signs, with the exception of intermittent regurgitations when fed a large food item. The snake lived up to 15 months after surgery, and although no postmortem examination was performed,* Cryptosporidium* sp.-associated chronic proliferative gastritis is considered the most likely cause of death.
